# Supplementary material for: Feasibility of motor imagery and effects of activating and relaxing practice on autonomic functions in healthy young adults: A randomised, controlled, assessor-blinded, pilot trial
Source: PLoS One. 2021 Jul 13;16(7):e0254666. doi: 10.1371/journal.pone.0254666 (PMC8277051; doi:10.1371/journal.pone.0254666)
Supplement: S1 File — (PDF) [file pone.0254666.s003.pdf]

S1 File. PETTLEP principles of the motor imagery interventions.

Participants were carefully instructed about their suggested physical **P**ositions during motor imagery (MI) practice, choice of a suitable, quiet training **E**nvironment, the **T**asks to be performed, the **T**iming of the imagined movements, how to enhance their MI experience (**L**earning), arousal related (group 1)/calmness and relaxation related (group 2) and motivational aspects of the MI tasks (**E**motion) and the above mentioned MI **P**erspective in their respective groups (PETTLEP approach[1]).

Position (Physical): Participants were instructed to practise at any time of the day when alert; to keep eyes open or closed, depending on the MI task and personal preference; to breath normally; to assume a body position as shown in the video, and hence consistent with the exercise to imagine; to relax one's shoulders, and avoid tightening any muscles or moving.

Environment: Participants were asked to practice in a quiet place at home; imagine practicing in a fitness studio or place which is suitable for a workout (group 1) or in a body-mind centre or place that is suitable for gentle and relaxing body movements (group 2); this was supported by the videos.

Tasks and Timing: The activating kinaesthetic MI training involved imagining high effort exercises: superman exercises; contralateral limb raises; jogging in place; squats and squat jumps; crunches; lunges; planking; side-lying hip abduction; air boxing; side to side jumping; glute bridge and push-ups. The timing was internal and depended on the tempo and intensity of the MI tasks. Both a high tempo and high intensity were used in group 1. For example, the MI script for planking was: *"I would like you to imagine yourself performing this front plank exercise while using all your available muscle power. Imagine keeping the position of the abdominal bridge for as long as possible while breathing normally. Perform the imagined exercise as powerfully as possible but avoid tightening any muscles during the motor imagery task."* A similar script was used with all tasks. Each exercise was imagined for around 60-90 seconds, alternated with a short video and description of the next MI task.

Contrastingly, the relaxing kinaesthetic MI training included imagined relaxing (low effort) exercises: hamstrings stretch in supine position; quadriceps stretches in side-lying position; pectoral stretch in supine position; pelvic tilt and contrary motion with head in supine position; chest stretch starting on all fours (child's pose); deep

abdominal breathing with hands on chest and stomach while seated; slow upper body and head rotation while seated; a sole movement of one foot from arch to knee of the other foot and vice versa; arm-raise while seated and during inspiration, arm drop during expiration using pursed-lip technique; self-massage of arms, chest, stomach, and legs with flat hands; comfortable arm-swing while seated and slight upper body rocking and full-body stretch while lying supine. The timing was internal and depended on the tempo and intensity of the MI tasks. A slow tempo and low intensity were used in group 2. For example, the MI script for the hamstrings or quadriceps stretch was: *"I would like you to imagine yourself performing this stretching exercise of the legs as slowly as possible. Imagine yourself moving as effortlessly as possible while consciously breathing very slowly and deeply. Imagine that with every breath, your body relaxes even deeper into the position"* A similar script was used with all tasks. Each exercise was imagined for around 60-90 seconds, alternated with a short video and description of the next MI task.

Learning: During the introductory MI sessions, participants were familiarised in small groups of 2 or 3 individuals with the MI modes (kinaesthetic and visual) and perspectives (internal and external) and the types of MI tasks designed for their respective study group by the researcher (TK). They could try out different modes and perspectives and find out their preferences, however the use of a kinaesthetic mode was stressed. Individuals were informed about how to enhance their MI ability and experience (Learning).

Emotion: The MI instructions included motivational and arousal modifying aspects. In group 1, overall, due to the high intensity of the imagined exercises, the MI instructions aimed at producing a strong sense of power, self-confidence, energy, and high arousal. In group 2, generally, due to the low intensity and focus on deep breathing, the MI instructions aimed at creating a perception of ease, relaxation, and well-being.

Perspective: As already mentioned, kinaesthetic MI from an internal, first-person perspective was preferably used.

References

1. Holmes PS, Collins DJ. The PETTLEP approach to motor imagery: A functional equivalence model for sport psychologists. Journal of Applied Sport Psychology. 2001;13(1):60-83.
