# Supplementary material for: Feasibility of motor imagery and effects of activating and relaxing practice on autonomic functions in healthy young adults: A randomised, controlled, assessor-blinded, pilot trial
Source: PLoS One. 2021 Jul 13;16(7):e0254666. doi: 10.1371/journal.pone.0254666 (PMC8277051; doi:10.1371/journal.pone.0254666)
Supplement: S2 File — (PDF) [file pone.0254666.s004.pdf]

S2 File. Qualitative methods and results.

Interviews

Prior to the interviews, the Master's course leader (study PI, physiotherapist, PhD) provided a formal training programme. This was followed by practice interviews where the students interviewed their course leader. In addition, using the interview guide, pilot interviews were undertaken in fellow students. The first two formal interviews of the study participants were supervised by the course leader. Nobody else was present at the interviews. Interviewers were identical to the blinded raters and not aware of the participants' group allocations until the start of the interview. Initially, interviewers introduced themselves to the participants. Interviews were taken down simultaneously by one of the interviewers and additional field notes were made by the other one (duration 20-30 minutes). Interview transcripts were returned to the participants for comment and/or correction. In S1 Table 1, the pre-defined questions of the semi-structured interview are shown.

Qualitative data analysis

Data were organised based on the research question. Reoccurring ideas, concepts, words and phrases were identified and scrutinised. Based on that, a coding frame was developed to group them into meaningful categories. Requirements for categories and subcategories were their mutual exclusiveness and exhaustiveness, apparent one-dimensionality and saturation, where each subcategory is used at least once. Relevant material was selected and text segments structured and generated, marked and defined, subdivided, revised and expanded and central subcategories identified, based on the MI theory (1, 2). Categories were defined, named, and characterised and decision rules defined for any cases of overlapping subcategories, to allow for a consistent assignment of data segments. The coding frame was tried out, revised and

expanded according to the data as appropriate. The material was progressively summarised, subsumed and contrasted. Categories and subcategories were illustrated using citations. This was followed by creating a data matrix suitable for quantitative data analysis. Descriptive statistics (frequencies) were employed.

Results

Seven major themes emerged from the analysis, which are listed together with relevant categories as follows.

- MI tasks: greater ease of MI of movements which one is familiar with and capable of performing in real; body parts concerned: it is more difficult to imagine trunk movements than extremity movements; MI of changing and maintaining certain body positions depends on one's physical abilities.
- MI modes: visual, kinaesthetic, or mixed; references to visual MI: watching oneself; seeing oneself moving; references to kinaesthetic MI: feeling that muscles want to contract; perception of increased heartbeat frequency; sensing the movement very naturally although it is not being executed; feeling of a generally enhanced body perception.
- MI perspectives: internal (watching/feeling oneself moving and seeing the environment as usually) and external (watching oneself from the outside); perception of an overall increased visual imagination.
- Training related factors: training material; number and variation of MI tasks; progression of training difficulty; length and detail of task instructions; length of pauses between tasks (should be rather short); and therapist supervision (wished).
- Barriers to MI: fatigue; loss of concentration; the novelty of the training; distracting thoughts.

- Facilitators to MI: closed eyes; calm environment; being alone; learning effect (MI ability improves with practice).
- Subjective MI effects: MI practice increases or decreases fatigue; enhances mood; relaxes; is (not) exhausting; feeling of calmness, comfort, and relaxation; feeling disburdened from stressors; being surprised by the newness of the MI experience; wellbeing induced by MI.

Participants expressed their experiences with MI [of planks] and referred to reduced MI ability if they were not able to carry out a particular exercise in real. They also described fatigue as a hindrance to concentration (group 1).

“Some exercises were easy to do. Making one was particularly difficult. Because it was a move I couldn't normally perform, and I always focus on what I can't do. It was easy to make the starting position, but I had trouble coming back while making the descents of the movement.” (ID 40, group 1)

“I had some troubles focusing because of tiredness, so it was hard to imagine when I was tired. (ID 15, group 1)

Participants in group 2 also raised the matter of familiarity with motion tasks. They also referred to their perception of relaxation that was induced by the MI:

“It was easy when the environment was quiet, so my focus was good. Because I saw the movements before [on the videos] and it was easy to imagine since I had experienced them [in real] in my previous life.” (ID 19, group 2)

“The content of the motor imagery was good. Even though I was not doing sports, feeling that I do those movements relaxed me.” (ID 36, group 2)

When asked for their MI perspectives, participants expressed the following:

“During the MI, I imagined the surroundings very close to reality.” (ID 53, group 1)

“When I focused [on the motor imagery], the sensation of the body area I focused on increased and sometimes I felt as if my muscles were contracting.” (ID 54, group 2)

When interviewed whether or not they would like to continue with the MI after the study and why, all but three participants expressed their interest in continuing. The main barrier for one of the participants was time, another one was disinterest and reasons number 3 and 4 are cited below.

“I would like to continue. Because as the number of repetitions will increase with a longer period, it is interesting to imagine the movements and to see the plasticity effect accordingly.” (ID 40, group 1)

“I do not intend to continue as it does not cause any meaningful change in my life.” (ID 19, group 2)

All participants mentioned that they would recommend practising MI to somebody else, for several reasons:

“I think that being able to imagine increases the ability to feel the movement I make. Therefore, I recommend it.” (ID 15, group 1)

“Excessive fatigue may occur while doing the [real activating] exercises. This is not the case during motor imagery so I can recommend it to others. And I would recommend it as it provides calmness and relaxation as you get away from daily thoughts.” (ID 30, group 2)

References

1. Jeannerod M. The representing brain: neural correlates of motor intention and imagery. Behav Brain Sci. 1994;17:187-202.
2. Holmes PS, Collins DJ. The PETTLEP Approach to Motor Imagery: A Functional Equivalence Model for Sport Psychologists. Journal of Applied Sport Psychology. 2001;13(1):60-83.
