# Supplementary material for: Feasibility of motor imagery and effects of activating and relaxing practice on autonomic functions in healthy young adults: A randomised, controlled, assessor-blinded, pilot trial
Source: PLoS One. 2021 Jul 13;16(7):e0254666. doi: 10.1371/journal.pone.0254666 (PMC8277051; doi:10.1371/journal.pone.0254666)
Supplement: S1 Study protocol — (PDF) [file pone.0254666.s005.pdf]

A. **NAME OF THE RESEARCH**

A.1	**SCIENTIFIC NAME OF THE RESEARCH**<br>Effects of motor imagery on autonomic function and motor imagery abilities in healthy individuals	
A.2	**SCIENTIFIC NAME OF THE RESEARCH IN ENGLISH**<br>Effects of motor imagery on autonomic function and motor imagery abilities in healthy individuals	
A.3	**PUBLIC NAME OF THE RESEARCH**<br>Investigation of the effects of two different kind of motor imagery on autonomic function and imagery abilities in healthy adults	
A.3.1	**CHARACTER OF THE RESEARCH**	
A.3.2	Master thesis/project	☐
A.3.3	Doctoral thesis	☐
A.3.4	MD thesis	☐
A.3.5	Individual Research project	☒
A.3.6	The short name of research	☐
A.3.7	Other	☐

C. **INFORMATION ABOUT THE RESEARCH**

C.1

INTRODUCTION

Motor imagery is defined as thinking the movement mentally without revealing a real movement (Sharma, Pomeroy, & Baron, 2006). Most of the current physiotherapy and rehabilitation approaches are based on real movements to stimulate damaged motor neural connections through neuroplasticity (Kleim, 2011).

Studies have shown that similar brain regions are activated during motor imagery and real movement (Gerardin et al., 2000; Kraeutner, Gionfriddo, Bardouille, & Boe, 2014). By imagining the same movement repeatedly, people can improve their motor activity skills such as lifting weights, playing the piano, or performing surgery (Schieber, 2013). These findings suggest that motor imagery provides motor learning by causing strengthening in synaptic connections depending on activity (Schieber, 2013). Mirror neurons that are activated while watching the movements of others are also activated during motor imagery and play a key role in neuroplastic changes revealed by motor imagery (Schieber, 2013). In many studies conducted with both acute and chronic stroke patients, Parkinson's patients and athletes, it has been reported that motor imagery training improves movement performance, and this change is associated with neuroplasticity (Mulder, 2007).

Unlike real movement-based methods, in motor imagery, the patient does not need to be able to perform the relevant movement. This enables motor imagery to act as a bridge to the transition to true movement-based therapy, especially in patients with severe physical dysfunction (Page, Levine, & Hill, 2007; Sharma et al., 2006). In addition, it provides the opportunity to work with higher doses compared to real movement therapy-based therapies and without the need for a special environment such as a clinic or various exercise equipment.

According to systematic reviews including studies on motor imagery training, it has been emphasized that motor imagery training can be a potential treatment method for stroke patients in improving motor activities and functions (Braun et al., 2013; Li, Li, Tan, Chen, & Lin, 2017). In a recent systematic review and meta-analysis, it was reported that motor imagery training increased muscle strength of healthy individuals, and it was emphasized that it was a method that should be added to the rehabilitation of athletes (Paravlic et al., 2018).

Generally, motor imagery is divided into two as visual and kinesthetic (Lim et al., 2006). During visual motor imagery, the individual imagines that he or she is watching the movement from a distance (third-person perspective), while during kinesthetic imagery she/he imagines that she/he is doing the movement (first-person perspective) (Sirigu &

	Duhamel, 2001). Motor imagery can also be defined as implicit or explicit (de Vries et al., 2013). Explicit motor imagery is defined as a phenomenological experience in which the sense of movement is experienced consciously (de Vries et al., 2013). That is, the individual is aware that he is imagining movement. On the other hand, implicit motor imagery is defined as unconscious visualization of movement (de Vries et al., 2013). For example, when an individual looks at a photograph of a hand standing in a different position than normal, he unwittingly imagines his hand gestures moving the hand to the position he is used to and decides whether the hand in the photograph is right or left. Open and closed motor visualization describe different aspects of cognitive task and should not be considered as separate situations from each other. A motor imagery task can be performed both visually and off, kinesthetic, and open, or vice versa (McInnes, Friesen, & Boe, 2016). However, motor imagery tasks used in neurorehabilitation are mostly explicit motor imagery, utilizing both kinesthetic and visual motor imagery (McInnes et al., 2016).
C.2	**RATIONALE/PURPOSE OF STUDY**<br><br>Most of the studies on motor imagery attribute its mechanism of action to neuroplastic changes occurring in the brain (Gerardin et al., 2000; Kraeutner et al., 2014; Mulder, 2007; Schieber, 2013). On the other hand, it is thought that motor imagery can alter metabolic responses, just like in real exercise. In a study conducted on this subject, it was reported that physiological changes such as heart rate, oxygen consumption, blood pressure, respiratory rate, metabolic rate after motor imaging were like actual exercise (Wang & Morgan, 1992). However, the level of evidence for the effect of motor imagery on autonomic functions is extremely limited.<br><br>The aims of this study are;<br><br>1. To examine the effects of the activating and relaxing kinesthetic motor imagery training program on autonomic functions in healthy individuals and to compare these two methods with each other.<br><br>2. In the study, the effects of the mentioned training program on the motor imagery skills of individuals will also be examined.
C.3	**MATERIAL – METHOD**<br><br>**Sample size**<br><br>According to Julious (Julious, 2005), 12 participants per group is considered the minimum sample size for a pilot study (Julious, 2005). Treece and Treece suggested that for pilot studies, 10% of the total sample size required for a full-scale study should be

taken (Treece & Treece, 1982). Based on results from a previous motor imagery training study using physiological measurements (mean and standard deviation of heart rate differences between groups), the sample size of 63 participants per group was obtained using the HyLown sample size calculation software (http://powerandsamplesize.com/). Since there will be three groups in this study, it was determined that 189 participants were needed for a full-scale study. Based on these recommendations in the literature, it was decided that the sample size for this pilot study should be 15 participants per group. The total sample size was planned as 54 participants (18 per group), with the addition of possible losses of 20%.

Randomization

Participants will be divided into three groups using a simple randomization method based on a single sequence using computer software (https://www.randomizer.org/) by a researcher who will not be involved in evaluation and treatment (Altman & Bland, 1999).

Motor imagery training

DVDs containing motor imagery instructions will be given to the individuals participating in the motor imagery training. The same content will be made available for download online to participants (control) who wish to do so.

Group 1 will be trained in activating kinesthetic motor imagery. Scenarios in this motor visualization training will focus on exercises that require high effort (plank, boxing, jumping, crouching, push-up, etc.). In the motor imagery scenarios of the participants in Group 2, low intensity (relaxation) exercises (breathing exercises, stretching, body awareness exercises, etc.) will be included. Motor imagery training for both groups, 5 days a week, 17 minutes a day. It will be applied for 2 weeks (Schuster et al., 2011).

C.3.1	**TYPE / SUBJECT / DESIGN OF THE RESEARCH**	
C.3.1.1	Observation studies	☒
C.3.1.2	Survey studies	☐
C.3.1.3	Retrospective archive scans such as file and image records	☐
C.3.1.4	Research with biochemistry, microbiology, pathology, and radiology collection materials such as blood, urine, tissue, radiological images	☐

C.3.1.5	Research to be made with materials obtained during routine examination, examination, analysis, and treatment processes	☐
C.3.1.6	In vitro studies with cell and tissue cultures	☐
C.3.1.7	Research to be conducted with genetic material for identification purposes other than gene therapy clinical trials	☐
C.3.1.8	Research to be conducted within the boundaries of nursing activities	☐
C.3.1.9	Diet studies include food additive	☐
C.3.1.10	Body physiology studies like exercise	☒
C.3.1.11	Lifestyle assessment studies	☐
C.3.1.12	Survey studies	☐
C.3.1.13	Please note if other (neurologic data for using human and social sciences)	☐
C.3.2	**RESEARCH CENTER**<br>Izmir Katip Celebi University, Faculty of Health Sciences, Physiotherapy and Rehabilitation, Research Laboratories	
C.3.3	**CHARACTERISTICS OF THE VOLUNTEER GROUP**	
C.3.3.1	Estimated number of volunteers	54
C.3.3.2	Child (under 18 years old)	☐
C.3.3.3	Adult (+18 years old)	☒
C.3.3.4	Woman	☒
C.3.3.5	Man	☒
C.3.3.6	Healthy participant	☒

C.3.3.7	Patient	☐
C.3.3.8	Disabled person	☐
C.3.3.9	Pregnant	☐
C.3.3.10	Breastfeeding woman	☐
C.3.3.11	Emergency patient	☐
C.3.3.12	Students	☒
C.3.3.13	Older people	☐
C.3.3.14	Another situation	☐
C.3.4	**INCLUSION AND EXCLUSION CRITERIA**<br>Inclusion criteria<br>1. Being healthy (not having any known, diagnosed chronic diseases)<br>2. Volunteering to participate in the study<br>Exclusion criteria<br>1. Presence of orthopedic or neurological disease<br>2. Pregnancy	
C.3.5	**CONTROL GROUP**<br>Participants to be included in Group 2 and Group 3 will be matched by age, gender and dominant hand with those in Group 1.	
C.3.6	**THE PLACE THE RESEARCH WILL BE CONDUCTED AND PERMISSIONS**<br>The research will be done in the laboratory of Izmir Katip Celebi University Faculty of Health Sciences, Physiotherapy and Rehabilitation under the permission of the Head.	
C.3.7	**DATA COLLECTION TOOLS AND FEATURES**<br>**Dominant hand preference**	

The Edinburgh Handedness Questionnaire (Oldfield, 1971) will be used to determine the dominant hand preference. In this questionnaire, hand preference used in different activities is questioned. The Turkish version of the questionnaire has been shown to be valid and reliable (Nalcaci, Kalaycioglu, Gunes, & Cicek, 2002).

Applicability (feasibility) of the methods

The feasibility of the methods (safety, side effects, rate of participant acceptance of applications), the feasibility of a full-scale study (target sample size, dropout rate, participation in targeted sessions) will be examined. The rate of falling out of the study should not be more than 20% and the participation in the sessions should not be less than 70%.

Measuring autonomic functions

Physiological responses as an indicator of autonomic function will be examined by measuring basal metabolic rate. A cardiopulmonary exercise test device will be used to measure physiological responses. For this purpose, Cosmed Quark CPET (Cosmed, Rome, Italy) branded device will be used in the Laboratory of Department Physiotherapy and Rehabilitation of Faculty of Health Sciences, İzmir Kâtip Çelebi University. This device provides a suitable mixing environment for gas exchange and instantaneous respiratory analysis (VO2, VCO2), 12-lead integrated electrocardiography monitoring, low and high ventilation ranges.

Each participant will be asked not to take food, caffeine, and nicotine at least 4 hours before. In addition, participants will be asked to avoid strenuous exercise 24 hours before the measurement (Vandarakis, Salacinski, & Broeder, 2013). The measurements will be taken while the participants are wearing comfortable clothing and lying on their backs on a stretcher. The room will be ventilated at least 1 hour before measurement and the room temperature will be kept at 20-23°C. Before measurement, the system will be calibrated to ensure that the O_2 and CO_2 gas concentrations are suitable for the test. All measurements will be taken in the morning.

During the measurement, the participant is put on a mask designed to contain his mouth and nose and will be asked to lie in bed for 15 minutes without doing anything. The system will automatically record data about the participant's metabolic expenditure at rest. These data are divided into three as metabolic, respiratory, and spent resources. Metabolic variables; caloric expenditure (RMR), oxygen uptake (VO_2), carbon dioxide production (VCO_2). Respiratory variables: ventilation (VE) is FeO_2 and $FeCO_2$. The variables about the resources spent are fat, carbohydrate and protein.

	**Measures of motor imagery ability**<br><br>1. Movement Imagery Questionnaire-Revised is a motor imagery questionnaire used in healthy adult and athletic populations and includes movements that require a high degree of skill and coordination (Butler et al., 2012). The visual and kinesthetic motor imagery skills of the person are measured with the scale consisting of 8 items. Items related to visual imagery are evaluated using a Likert-type scale between 1 (very difficult to see) and 7 (very easy to see). Items related to kinesthetic imagery are likewise evaluated using a Likert-type scale that is graded between 1 (very difficult to feel) and 7 (very easy to feel). High scores indicate high visual and kinesthetic imagery skills. The Turkish validation study of the questionnaire was conducted, and it was shown to be valid and reliable (Akkarpat, 2014).<br><br>2. Motor imagery skills will also be evaluated using a mental chronometry paradigm. The mental chronometry is based on measuring the temporal harmony between a real movement and the imagination of the same movement. Mental chronometry assessment will be applied for both lower and upper extremity movements. 6 m walking test will be applied for lower extremity. A 6 m line will be drawn on the ground with a mark at the beginning and end. Participants will be asked to imagine that they are walking this line at their normal speed from a first-person perspective (as if they were doing it themselves). Then the 6 m walking test will be applied in the form of real motion. Upper extremity of the participants for mental stopwatch test "Türkiye'nin başkenti Ankara" really leave behind you will be asked to imagine that they wrote the sentence. Participants will hold a digital stopwatch in their non-dominant hands during all tests and will start and stop time at the end of both real and imagined movement (Papaxanthis, Pozzo, Skoura, & Schieppati, 2002).<br><br>3. Hand lateralization judgment test<br><br>During this test, the participant is asked to indicate which hand shapes shown belong to the right or left hand. The high number of correct numbers is considered to be an indicator of high motor imagination (Boonstra et al., 2012). In this study, a tablet software (Recognise ™ Flash Cards, NOI) with proven validity and reliability will be used (Zimney et al., 2018). During the test, different hand photographs of the tablet screen appear, and the participant is asked to choose which hand (right or left) the corresponding picture is.
C.3.8	**OPERATIONS AND VARIABLES TO BE USED IN THE RESEARCH**<br>In this study, participants will be randomly assigned to one of three groups: Group 1: activating kinesthetic motor imagery training; group 2: relaxing kinesthetic motor

	imagery training; and group 3: control group. After randomization and before the implementation, participants will be given an introductory training to become familiar with motor imagery. The concept of motor imagery and its uses in sports and rehabilitation will be explained. Different types of motor imagery (kinesthetic and visual) and perspectives (inner, first-person and outer, third-person perspective) will be explained theoretically and practically. Participants will be encouraged to practice on their own and be made aware of their preferred type and perspective of motor imagery. Emphasis will be placed on the use of internal and kinesthetic motor imagery in this study. Introductory training before motor image training is recommended by various authors (Schuster et al., 2011; Wondrusch & Schuster-Amft, 2013).<br><br>The PETTLEP framework strategy based on a neuroscientific basis will be used for motor imagery. This strategy consists of Physical, Environmental, Task, Timing, Learning, Emotional and Perspective components (Holmes & Collins, 2001). PETTLEP is a method developed to increase performance in athletes (Holmes & Collins, 2001). PETTLEP elements include the practitioner's physical position according to the motor visualization task and perspective, the imagined environment, the imagined task, the timing of the motor imagery, learning or changes with motor imagery, and emotional states. PETTLEP ideas will be applied in this pilot study to support participants' understanding of the application and effectiveness.<br><br>After the familiarization training and verbal instructions, the participants will be given DropBox links containing engine visualization training videos specially designed for their groups. Participants will be able to download the engine imagination training videos and instructions and watch them on their smartphones, computers, or tablets via these links. Participants will be asked to apply the kinesthetic motor imagery training at their own homes, 5 times a week, once a day for 17 minutes. The videos will be used to enable participants to see the exercises to accurately visualize the relevant movements. The frequency and duration of motor imagery sessions were decided according to the results of a review examining motor imagery training applied in different disciplines (Schuster et al., 2011). After a week, the participants will be called by phone, support will be provided for the application and will be reminded that there will be a post-implementation evaluation. The frequency of the application will be recorded in their exercise logs and they will be asked to report it on the phone. However, there will not be a direct exercise follow-up. Participants in the control group will not be given a special application but will be called by phone to remind the time of the second evaluation.

C.3.9	**STATISTICAL ANALYSIS**<br><br>SPSS (version 24.0, IBM Corporation, Armonk, NY, ABD) software will be used in all statistical analyzes. The statistical significance level will be considered as two-sided p <0.05. The compliance of the data to normal distribution will be evaluated by examining the results of skewness and kurtosis and histograms in addition to the Shapiro-Wilk test results.<br><br>Intention-to-treat analysis will be performed for all participants with follow-up data, considering the groups they were originally assigned to. Descriptive statistics will be used to summarize initial demographic variables.<br><br>The significance of the difference between pre-and post-training data will be analyzed in the dependent group using the t-test / Wilcoxon signed-rank test in accordance with the given distribution characteristics. Bonferroni correction will be used where necessary. Two-factor mixed pattern analysis of variance (ANOVA) will be used to examine continuous variables such that groups are a factor between participants and time is a factor for participants. Effect sizes will be calculated as partial eta square values. When a meaningful result is obtained in the relevant analysis, the reason for the difference will be examined with appropriate post hoc correction methods.
C.3.10	**RESEARCH PERIOD**<br><br>**Research start date:** After the date of ethics committee approval.<br><br>**The estimated duration of the study:** 24 months.
C.3.11	**REFERENCES**<br><br>Altman, D. G., & Bland, J. M. (1999). Statistics notes. Treatment allocation in controlled trials: why randomise? BMJ, 318(7192), 1209.<br><br>Boonstra, A. M., de Vries, S. J., Veenstra, E., Tepper, M., Feenstra, W., & Otten, E. (2012). Using the Hand Laterality Judgement Task to assess motor imagery: a study of practice effects in repeated measurements. Int J Rehabil Res, 35(3), 278-280. doi: 10.1097/MRR.0b013e328355dd1e<br><br>Braun, S., Kleynen, M., van Heel, T., Kruithof, N., Wade, D., & Beurskens, A. (2013). The effects of mental practice in neurological rehabilitation; a systematic review and meta-analysis. Front Hum Neurosci, 7, 390. doi: 10.3389/fnhum.2013.00390<br><br>Butler, A. J., Cazeaux, J., Fidler, A., Jansen, J., Lefkove, N., Gregg, M., . . . Wolf, S. L. (2012). The Movement Imagery Questionnaire-Revised, Second Edition (MIQ-RS) Is a Reliable and Valid Tool for Evaluating Motor Imagery in Stroke Populations. Evidence-

based complementary and alternative medicine : eCAM, 2012, 497289-497289. doi: 10.1155/2012/497289

de Vries, S., Tepper, M., Feenstra, W., Oosterveld, H., Boonstra, A. M., & Otten, B. (2013). Motor imagery ability in stroke patients: the relationship between implicit and explicit motor imagery measures. Front Hum Neurosci, 7, 790. doi: 10.3389/fnhum.2013.00790

Gerardin, E., Sirigu, A., Lehericy, S., Poline, J. B., Gaymard, B., Marsault, C., . . . Le Bihan, D. (2000). Partially overlapping neural networks for real and imagined hand movements. Cereb Cortex, 10(11), 1093-1104.

Holmes, P. S., & Collins, D. J. (2001). The PETTLEP Approach to Motor Imagery: A Functional Equivalence Model for Sport Psychologists. J Appl Sport Psychol, 13(1), 60-83. doi: 10.1080/10413200109339004

Julious, S. A. (2005). Sample size of 12 per group rule of thumb for a pilot study. Pharm Stat, 4, 287-291. doi: 10.1002/pst.185

Kleim, J. A. (2011). Neural plasticity and neurorehabilitation: teaching the new brain old tricks. J Commun Disord, 44(5), 521-528. doi: 10.1016/j.jcomdis.2011.04.006

Kraeutner, S., Gionfriddo, A., Bardouille, T., & Boe, S. (2014). Motor imagery-based brain activity parallels that of motor execution: evidence from magnetic source imaging of cortical oscillations. Brain Res, 1588, 81-91.

Li, R. Q., Li, Z. M., Tan, J. Y., Chen, G. L., & Lin, W. Y. (2017). Effects of motor imagery on walking function and balance in patients after stroke: A quantitative synthesis of randomized controlled trials. Complement Ther Clin Pract, 28, 75-84. doi: 10.1016/j.ctcp.2017.05.009

Lim, V. K., Polych, M. A., Holländer, A., Byblow, W. D., Kirk, I. J., & Hamm, J. P. (2006). Kinesthetic but not visual imagery assists in normalizing the CNV in Parkinson’s disease. Clin Neurophysiol, 117(10), 2308-2314.

McInnes, K., Friesen, C., & Boe, S. (2016). Specific brain lesions impair explicit motor imagery ability: a systematic review of the evidence. Arch Phys Med Rehabil, 97(3), 478-489. e471.

Mulder, T. (2007). Motor imagery and action observation: cognitive tools for rehabilitation. J Neural Transm (Vienna), 114(10), 1265-1278. doi: 10.1007/s00702-007-0763-z

Nalcaci, E., Kalaycioglu, C., Gunes, E., & Cicek, M. (2002). [Reliability and validity of a handedness questionnaire]. Turk Psikiyatri Derg, 13(2), 99-106.

Oldfield, R. C. (1971). The assessment and analysis of handedness: the Edinburgh inventory. Neuropsychologia, 9(1), 97-113.

Page, S. J., Levine, P., & Hill, V. (2007). Mental practice as a gateway to modified constraint-induced movement therapy: a promising combination to improve function. Am J Occup Ther, 61(3), 321-327.

Papaxanthis, C., Pozzo, T., Skoura, X., & Schieppati, M. (2002). Does order and timing in performance of imagined and actual movements affect the motor imagery process? The duration of walking and writing task. Behav Brain Res, 134(1-2), 209-215.

Paravlic, A. H., Slimani, M., Tod, D., Marusic, U., Milanovic, Z., & Pisot, R. (2018). Effects and Dose-Response Relationships of Motor Imagery Practice on Strength Development in Healthy Adult Populations: a Systematic Review and Meta-analysis. Sports Med, 48(5), 1165-1187. doi: 10.1007/s40279-018-0874-8

Schieber, Marc H. (2013). Mirror Neurons: Reflecting on the Motor Cortex and Spinal Cord. Current Biology, 23(4), R151-R152. doi: https://doi.org/10.1016/j.cub.2013.01.004

Schuster, C., Hilfiker, R., Amft, O., Scheidhauer, A., Andrews, B., Butler, J., . . . Ettlin, T. (2011). Best practice for motor imagery: a systematic literature review on motor imagery training elements in five different disciplines. BMC medicine, 9, 75. doi: 10.1186/1741-7015-9-75

Sharma, N., Pomeroy, V. M., & Baron, J. C. (2006). Motor imagery: a backdoor to the motor system after stroke? Stroke, 37(7), 1941-1952. doi: 10.1161/01.STR.0000226902.43357.fc

Sirigu, A., & Duhamel, J. (2001). Motor and visual imagery as two complementary but neurally dissociable mental processes. J Cogn Neurosci, 13(7), 910-919.

Treece, E. W., & Treece, J. W. (1982). Elements of research in nursing (3rd ed.). St. Louis, MO: Mosby.

Vandarakis, D., Salacinski, A. J., & Broeder, C. E. (2013). A comparison of COSMED metabolic systems for the determination of resting metabolic rate. Research in sports medicine, 21(2), 187-194.

	Wang, Y., & Morgan, W. P. (1992). The effect of imagery perspectives on the psychophysiological responses to imagined exercise. Behavioural Brain Research, 52(2), 167-174.<br><br>Wondrusch, C., & Schuster-Amft, C. (2013). A standardized motor imagery introduction program (MIIP) for neuro-rehabilitation: development and evaluation. Front Hum Neurosci, 7, 477. doi: 10.3389/fnhum.2013.00477<br><br>Zimney, K. J., Wassinger, C. A., Goranson, J., Kingsbury, T., Kuhn, T., & Morgan, S. (2018). The reliability of card-based and tablet-based left/right judgment measurements. Musculoskeletal Science and Practice, 33, 105-109.
