# Supplementary material for: Feasibility of motor imagery and effects of activating and relaxing practice on autonomic functions in healthy young adults: A randomised, controlled, assessor-blinded, pilot trial
Source: PLoS One. 2021 Jul 13;16(7):e0254666. doi: 10.1371/journal.pone.0254666 (PMC8277051; doi:10.1371/journal.pone.0254666)
Supplement: S1 Table — (PDF) [file pone.0254666.s006.pdf]

S1 Table. Questions asked in the semi-structured interviews.

Question no	Question content
General introduction to the interview	
Main interview	
1	Tell me about your experiences during the motor imagery training.
2	Has it been easy or difficult to imagine the movements / exercises? - Why so? - What has been easy / difficult?
3	What have you seen or felt during the motor imagery?
4	What do you think about continuing motor imagery after the study?"
5	How should motor imagery be for you to continue with it after the study?
6	What do you think about recommending motor imagery to someone else?
Prompts used	
	Could you talk me through your answer in more detail?
	What were you thinking of when you answered this question?
	You seemed to be a little hesitant about answering this question? Was there something else on your mind?
	Do you have any further comments?
	If responses from participants are somewhat unclear, the interviewer asked, ‘Why so?’
Conclusion and thanks	
