# Supplementary material for: Feasibility of motor imagery and effects of activating and relaxing practice on autonomic functions in healthy young adults: A randomised, controlled, assessor-blinded, pilot trial
Source: PLoS One. 2021 Jul 13;16(7):e0254666. doi: 10.1371/journal.pone.0254666 (PMC8277051; doi:10.1371/journal.pone.0254666)
Supplement: S2 Table — (PDF) [file pone.0254666.s007.pdf]

S2 Table. Effect sizes of activating and relaxing motor imagery related to all outcomes.

Parameters	Activating MI vs. controls	Relaxing MI vs. controls	Activating vs. relaxing MI	Overall
**Motor imagery ability measures**				
MIQ-R vis[1]	0.052	0.042	<0.001	0.082
MIQ-R kin[1]	0.064	0.055	<0.001	0.058
MC walking (6MWT)[2]	0.087	0.131	0.005	0.110
MC Writing[2]	0.004	<0.001	0.005	0.004
RL discr time L[2]	0.003	0.030	0.080	0.038
RL discr time R[2]	0.077	0.001	0.043	0.052
RL discr acc L[2]	0.004	0.001	0.002	0.004
RL discr acc R[2]	0.055	0.004	0.035	0.053
**Autonomic function: cardiorespiratory function and metabolism measures**				
RMR[2]	0.089	0.008	0.125	0.087
BMR[2]	0.041	0.130	0.008	0.130
Fat metabol[2]	0.012	0.024	0.001	0.018
CHO metabol[2]	0.001	0.024	0.012	0.016
RQ[2]	0.025	0.003	0.007	0.015
VO_2[2]	0.097	0.007	0.129	0.091
VCO_2[2]	0.046	0.010	0.086	0.056
VE[2]	0.081	0.029	0.006	0.050
RF[2]	<0.001	0.001	0.001	0.001
FEO_2[2]	0.006	0.107	0.060	0.075
$FECO_2$[2]	0.018	0.175	0.125	0.134

BMI, body mass index; BMR, basal metabolic rate (kcal); CHO, metabol carbohydrate substrates used for energy metabolism (%); Fat %, body fat percentage; Fat metabol, fat substrates used for energy metabolism (%); FECO2, concentration of carbon dioxide in the exhaled gases (%); FEO2, concentration of oxygen in the exhaled gases (%); MC Walking (6MWT), mental chronometry using a 6-Metre Walk Test; MC Writing, mental chronometry using a writing task; MIQ-R kin, Motor Imagery Questionnaire-Revised, kinaesthetic subscale (median values); MIQ-R vis, Motor Imagery Questionnaire-Revised, visual subscale (median values); Muscle %, body muscle percentage; RF, respiratory frequency; RL discr time L/R, right-left discrimination time for the left/right hand; RL discrim acc L/R, right-left discrimination accuracy for the left/right hand; RMR, resting metabolic rate - caloric expenditure (kcal/day); RQ, respiratory quotient; VCO2, carbon dioxide production (ml/min); VE, minute ventilation (l/min); VO2, oxygen uptake (ml/min); Water %, body water percentage.

[1]Eta squared effect sizes (η^2) from Kruskal-Wallis test.

[2]Partial eta squared effect sizes (η_p^2) from a two-factor mixed analysis of variance (ANOVA).
