# Supplementary material for: Feasibility of motor imagery and effects of activating and relaxing practice on autonomic functions in healthy young adults: A randomised, controlled, assessor-blinded, pilot trial
Source: PLoS One. 2021 Jul 13;16(7):e0254666. doi: 10.1371/journal.pone.0254666 (PMC8277051; doi:10.1371/journal.pone.0254666)
Supplement: Attachment — Submitted filename: Response to Reviewers.docx [file pone.0254666.s008.docx]

PONE-D-21-06610R1
Feasibility of motor imagery and effects of activating and relaxing practice on autonomic functions in healthy young adults: a randomised, controlled, assessor-blinded, pilot trial
PLOS ONE

Reviewers' comments:

Reviewer's Responses to Questions

**Comments to the Author**

1. If the authors have adequately addressed your comments raised in a previous round of review and you feel that this manuscript is now acceptable for publication, you may indicate that here to bypass the “Comments to the Author” section, enter your conflict of interest statement in the “Confidential to Editor” section, and submit your "Accept" recommendation.

Reviewer #1: All comments have been addressed

Reviewer #2: All comments have been addressed

Reviewer #3: (No Response)

2. Is the manuscript technically sound, and do the data support the conclusions?

The manuscript must describe a technically sound piece of scientific research with data that supports the conclusions. Experiments must have been conducted rigorously, with appropriate controls, replication, and sample sizes. The conclusions must be drawn appropriately based on the data presented.

Reviewer #1: Yes

Reviewer #2: Yes

Reviewer #3: Partly

________________________________________

3. Has the statistical analysis been performed appropriately and rigorously?

Reviewer #1: Yes

Reviewer #2: Yes

Reviewer #3: (No Response)

________________________________________

4. Have the authors made all data underlying the findings in their manuscript fully available?

The PLOS Data policy requires authors to make all data underlying the findings described in their manuscript fully available without restriction, with rare exception (please refer to the Data Availability Statement in the manuscript PDF file). The data should be provided as part of the manuscript or its supporting information, or deposited to a public repository. For example, in addition to summary statistics, the data points behind means, medians and variance measures should be available. If there are restrictions on publicly sharing data—e.g. participant privacy or use of data from a third party—those must be specified.

Reviewer #1: Yes

Reviewer #2: Yes

Reviewer #3: Yes

________________________________________

5. Is the manuscript presented in an intelligible fashion and written in standard English?

PLOS ONE does not copyedit accepted manuscripts, so the language in submitted articles must be clear, correct, and unambiguous. Any typographical or grammatical errors should be corrected at revision, so please note any specific errors here.

Reviewer #1: Yes

Reviewer #2: Yes

Reviewer #3: Yes

________________________________________

6. Review Comments to the Author

Please use the space provided to explain your answers to the questions above. You may also include additional comments for the author, including concerns about dual publication, research ethics, or publication ethics. (Please upload your review as an attachment if it exceeds 20,000 characters)

Reviewer #1: I am happy with the changes the authors have made and their responses to the points I previously raised.

Reviewer #2: thanks alot for your response

Reviewer #3: Table 2 Fat2 metabol2: typo 60.2 48.4-71.9)

Line 257, Line 321-322, Line Line 330-331, Table 2 footnote: for FeO2, FeCO2, VCO2, VO2, 2 to be subscripted.

**Authors’ response to the Reviewers**

Dear Reviewer #1,

Thank you for accepting our changes to the manuscript and responses.

Dear Reviewer #2,

Thank you for accepting our changes to the manuscript and responses.

Dear reviewer #3,

Thank you again for your comments on our manuscript and response. Please find our point-by-point response as follows.

Table 2 Fat2 metabol2: typo 60.2 48.4-71.9)

We have added the bracket so that it now reads 60.2 (48.4-71.9)

Line 257, Line 321-322, Line Line 330-331, Table 2 footnote: for FeO2, FeCO2, VCO2, VO2, 2 to be subscripted.

We did accordingly.
